# Conditional Mitigation of Dental-Composite Material-Induced Cytotoxicity by Increasing the Cure Time

**DOI:** 10.3390/jfb14030119

**Published:** 2023-02-22

**Authors:** Takanori Matsuura, Keiji Komatsu, Kimberly Choi, Toshikatsu Suzumura, James Cheng, Ting-Ling Chang, Denny Chao, Takahiro Ogawa

**Affiliations:** 1 Division of Regenerative and Reconstructive Sciences and Weintraub Center for Reconstructive Biotechnology, UCLA School of Dentistry, Los Angeles, CA 90095, USA; 2 Department of Periodontology, Graduate School of Medical and Dental Sciences, Tokyo Medical and Dental University, Tokyo 113-8510, Japan

**Keywords:** composite, light-curing, curing time, cytotoxicity, fibroblast

## Abstract

Light-cured composite resins are widely used in dental restorations to fill cavities and fabricate temporary crowns. After curing, the residual monomer is a known to be cytotoxic, but increasing the curing time should improve biocompatibility. However, a biologically optimized cure time has not been determined through systematic experimentation. The objective of this study was to examine the behavior and function of human gingival fibroblasts cultured with flowable and bulk-fill composites cured for different periods of time, while considering the physical location of the cells with regard to the materials. Biological effects were separately evaluated for cells in direct contact with, and in close proximity to, the two composite materials. Curing time varied from the recommended 20 s to 40, 60, and 80 s. Pre-cured, milled-acrylic resin was used as a control. No cell survived and attached to or around the flowable composite, regardless of curing time. Some cells survived and attached close to (but not on) the bulk-fill composite, with survival increasing with a longer curing time, albeit to <20% of the numbers growing on milled acrylic even after 80 s of curing. A few cells (<5% of milled acrylic) survived and attached around the flowable composite after removal of the surface layer, but attachment was not cure-time dependent. Removing the surface layer increased cell survival and attachment around the bulk-fill composite after a 20-s cure, but survival was reduced after an 80-s cure. Dental-composite materials are lethal to contacting fibroblasts, regardless of curing time. However, longer curing times mitigated material cytotoxicity exclusively for bulk-fill composites when the cells were not in direct contact. Removing the surface layer slightly improved biocompatibility for cells in proximity to the materials, but not in proportion to cure time. In conclusion, mitigating the cytotoxicity of composite materials by increasing cure time is conditional on the physical location of cells, the type of material, and the finish of the surface layer. This study provides valuable information for clinical decision making and novel insights into the polymerization behavior of composite materials.

## 1. Introduction

Light-cured composite resin materials are tooth-colored materials widely used in dental restorative treatment to fill cavities and fabricate temporary crowns [1,2]. They are mainly composed of inorganic filler, photoinitiator, and matrix monomer such as bisphenol A glycidyl methacrylate (bis-GMA) and urethane dimethacrylate (UDMA) [3,4,5]. Depending on the ratio of components, they are classified into flowable composites, which have low viscosity, and bulk-fill composites, which have high viscosity. These different properties make them suitable for different purposes [6,7].

The polymerization reaction occurs when the photoinitiator is activated by visible-light wavelengths between 450 and 490 nm, followed by the generation of free radicals and the formation of polymers [8]. Like other polymer-based materials, their chemical composition alters the biological properties and responses of soft tissues [9,10,11,12,13,14,15,16,17,18]. Many studies have reported cytotoxicity of monomer components, which varies with chemical composition, type, and amount of residual monomer leached [19,20,21,22,23,24,25,26,27,28,29,30,31]. In addition, the free radicals generated during and after polymerization cause significant cellular damage [11,13,14,15,32,33]. Even after photo-polymerization, bis-GMA is eluted as residual monomer for over a month [34]. It is also known that light-cured composites do not polymerize in areas in contact with oxygen, with an unpolymerized layer formed at the surface [35].

Since composite materials are often used in direct contact with, or close to, the gingival tissue, their cytocompatibility is important. Nevertheless, materials are usually selected based on user preference, user friendliness, or esthetics. Cytocompatibility is almost never considered when selecting the material.

For many composite materials, the manufacturers recommend a cure time of 20 s, but it is unclear whether this is biologically sufficient. Both clinically and theoretically, extending the cure time is expected to improve cytocompatibility by reducing the amount of residual monomer. A few studies have reported that extending the cure time improves cytocompatibility by reducing residual monomer [24,36]. However, the optimized cure time from the biological perspective has not been determined through systematic experimentation.

We have established an experimental method that simultaneously evaluates the behavior of cells in contact with, and close to, the test material [37,38]. Exploiting this approach, here we examined the behavior and function of human gingival fibroblasts cultured with two different composite materials (flowable and bulk-fill) cured for different periods of time, with a consideration of the physical location of the cells in relation to the materials: in direct contact with, and in close proximity to, each material. In addition, the effect of removing the unpolymerized layer on cytocompatibility was investigated. The null hypotheses are that cytocompatibility of composite materials is dependent on the curing time and the removal of the unpolymerized layer significantly reduces the adverse effects on fibroblasts.

## 2. Materials and Methods

### 2.1. Material Preparation and Characterization

**Figure 1:**
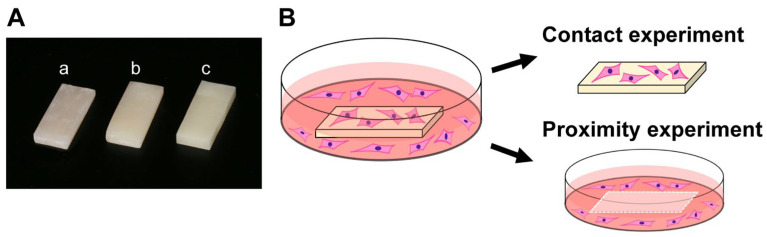
Test materials and culture-experiment design. (**A**) Test rectangular plates made of flowable composite (a), bulk-full composite (b), milled acrylic (c). (**B**) Contact and proximity experiments were performed separately, to mimic cellular reactions in vivo.

Flowable and bulk-fill composites were prepared in rectangular plate form (6 mm × 14 mm, 2 mm thickness) for evaluation (Figure 1A). Four different curing times were tested: 20, 40, 60, and 80 s. A light curing device (Coltolux LED; Coltène, Altstätten, Switzerland) was used to polymerize the samples with a wavelength of 450–470 nm and an intensity of 1275 mW/cm^2^. Milled-acrylic plates were designed using CAD software (123D Design, Hyperdent^®^, Synergy Health, Sydney, Australia) and manufactured from poly(methyl methacrylate) (PMMA) disks with a milling machine (Versamill 5 × 200, Axsys Dental Solutions, Wixom, MI, USA). These materials and their principal constituents are shown in Table 1 and Figure 1A. After preparation, all plates were washed with a steam cleaner and disinfected with 75% ethanol. Each test plate was placed on the center of each culture well, to standardize the physical distance between the plate and the cells in close proximity to the plate.

### 2.2. Cell Culture

Human gingival fibroblasts were purchased from ScienCell Research Laboratories (Carlsbad, CA, USA) and grown in fibroblast medium supplemented with 5% fetal bovine serum (FBS), 1% fibroblast growth supplement-2, and 1% penicillin/streptomycin solution. At 80% confluence, the cells were detached using 0.05% trypsin-EDTA solution, and seeded onto culture plates. Passage 5–8 cells were seeded onto test material placed in each well (20 mm diameter) of 12-well culture plates at a density of 4 × 10^4^ cells/well. The culture medium was renewed every three days. The UCLA Institutional Biosafety Committee (BUA-2-22-036-001) approved the study protocol.

### 2.3. Quantification of Attached and Propagated Cells

The number of attached fibroblasts was counted, to quantify the contact effect and proximity effect. The contact effect was defined as the quantification of fibroblasts attached to test materials, while the proximity effect was defined as the quantification of fibroblasts attached to the well of the culture dish around the material (Figure 1B). Attached fibroblasts were measured two days after seeding, and propagated fibroblasts were measured four and six days after seeding. The water-soluble tetrazolium salt (WST-1)-based colorimetric assay was used to quantify the number of cells, as reported elsewhere [39,40]. The amount of formazan product was measured at an absorbance of 450 nm, using a microplate reader (Synergy H1, BioTek Instruments, Winooski, VT, USA).

### 2.4. Fluorescent Microscopy

The cell structure on and around test materials was visualized by fluorescence microscopy (DMI6000B, Leica Microsystems, Wetzlar, Germany) two days after seeding. For this experiment, fibroblasts were cultured on glass-bottom 35 mm dishes, to stain cells around the test materials. Fibroblasts were dual stained with fluorescent dyes: 4′,6-diamidino-2-phenylindole (DAPI) to identify nuclei, and rhodamine-phalloidin for actin filaments. Fibroblast density was quantified by counting the cells in the images.

### 2.5. Collagen Production

Fibroblast collagen production was determined by Picrosirius-red staining (Picrosirius Red Staining Kit, Polysciences Inc., Warrington, PA, USA). As reported elsewhere [41,42,43], Picrosirius red stains collagen by reacting with basic groups present in the collagen molecule, via its sulfonic acid groups. Four days after seeding, cells were fixed in 10% formaldehyde. After binding Picrosirius red to produced collagen, 0.1 N sodium hydroxide was added and left for 60 min, to elute the binding dye. Then, the supernatant was measured at an absorbance of 550 nm, using a microplate reader.

### 2.6. Statistical Analysis

All cell-culture experiments were conducted in triplicate (*n* = 3). Results are expressed as mean ± standard deviations (SD). The test materials were compared using one-way analysis of variance (ANOVA) followed by the Tukey–Kramer *post hoc* test. *p*-values less than 0.05 were deemed statistically significant.

## 3. Results

### 3.1. Initial Cell Attachment

**Figure 2:**
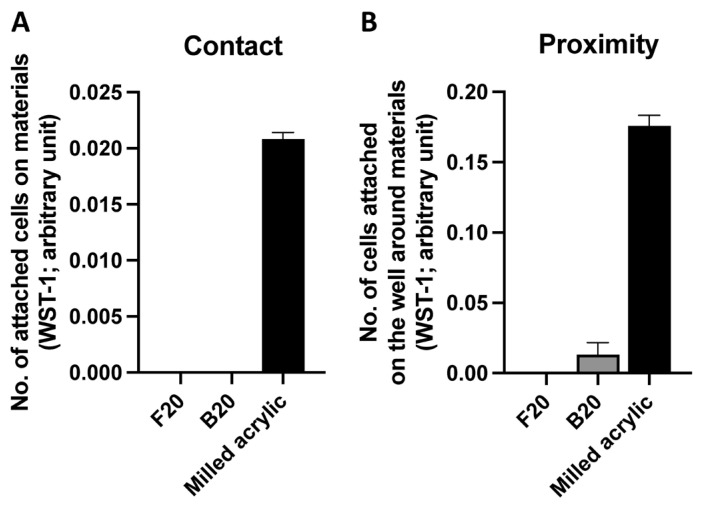
Initial attachment of fibroblasts on and around test materials cured for the manufacturer’s recommended time of 20 s. The number of attached fibroblasts (**A**) on each material (contact experiment) and (**B**) around each material (proximity experiment). Data shown are mean ± SD.

To evaluate the successful settlement of human fibroblasts after seeding, fibroblasts attached on or around the test material were counted, using the WST-1 assay, two days after seeding. There was no cell attachment in the contact experiment for either composite cured at 20 s, the manufacturer’s recommended time (Figure 2A). Similarly, in the proximity experiment, no cells attached around the flowable composite. Some fibroblasts attached around the bulk-fill composite, but only <10% of the number attaching around the milled-acrylic controls (Figure 2B).

**Figure 3:**
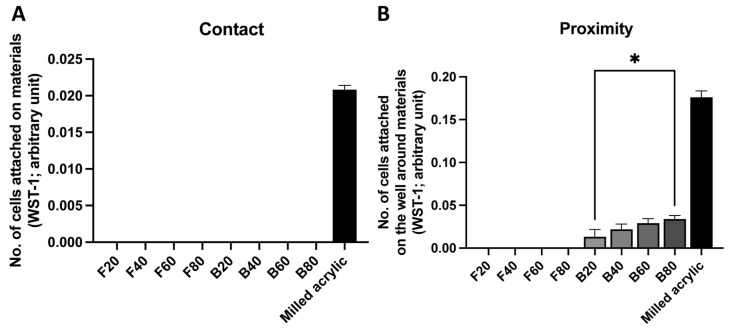
Initial attachment on and around test materials cured for different amounts of time. The number of attached fibroblasts (**A**) on each material and (**B**) around each material. Data shown are mean ± SD. Significant differences between groups are shown (one-way ANOVA followed by Tukey’s post hoc test, * *p* < 0.05).

Next, both materials were cured for 40, 60, or 80 s, and the same assay performed. No cells attached to either composite, regardless of the cure time in contact experiments (Figure 3A). As in the contact experiment, no cells attached around the d flowable composite in the proximity experiment at any timepoint. However, some cells attached around the bulk-fill composite, with the number increasing with a longer cure time, although the number was <20% of the number of cells attaching around the milled acrylic after 80 s of curing (Figure 3B). The number of cells around B80 was approximately three times higher than that of B20 (*p* < 0.05). The number of cells around B20, B40, B60, B80 and the milled acrylic were 0.013 ± 0.009, 0.022 ± 0.006, 0.029 ± 0.005, 0.034 ± 0.004 and 0.161 ± 0.012 arbitrary units, respectively.

### 3.2. Cell Proliferation

**Figure 4:**
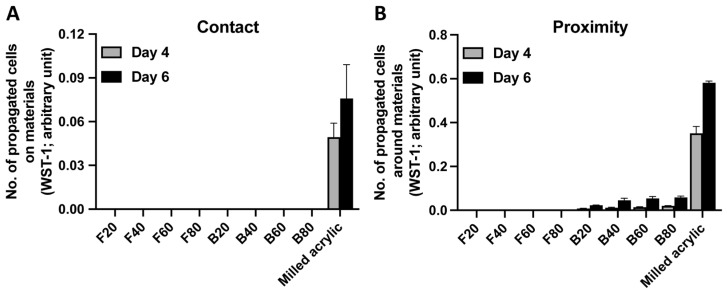
Propagation of fibroblasts on and around materials cured for different amounts of time. The number of propagated fibroblasts (**A**) on each material and (**B**) around each material. Data shown are mean ± SD.

Cell proliferation was evaluated by measuring the number of propagated cells four and six days after seeding. No cells attached to either composite on day 4 (Figure 4A). There were no propagated cells around flowable composite in the proximity experiment at either timepoint. The number of cells propagating around bulk-fill composite increased with cure time, although to levels < 15% of milled-acrylic controls (Figure 4B). The number of propagated cells around B20, B40, B60, B80 and milled acrylic were 0.023 ± 0.001, 0.046 ± 0.009, 0.055 ± 0.009, 0.059 ± 0.006 and 0.582 ± 0.008 arbitrary units, respectively.

### 3.3. Cell Visualization

**Figure 5:**
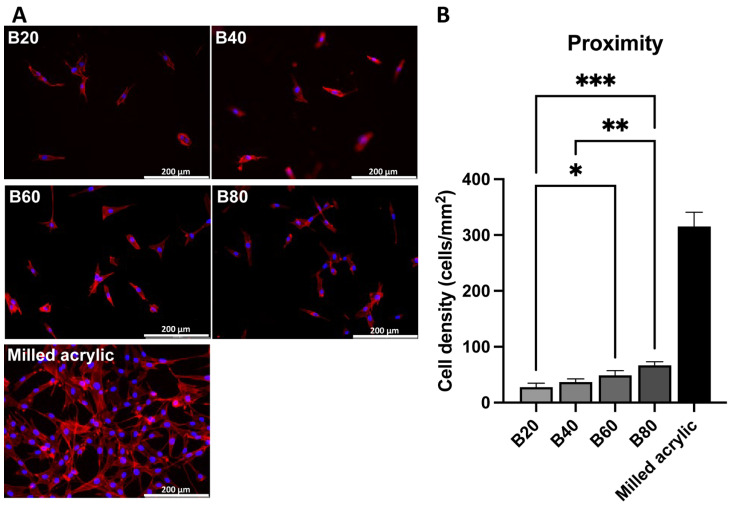
Visualized fibroblasts around test materials 2 days after seeding. (**A**) Fluorescent microscopic images of fibroblasts stained for nuclei (blue) and cytoskeletal actin filaments (red). (**B**) Cell density quantified in these images. Data shown are mean ± SD. Significant differences between test materials are shown (one-way ANOVA followed by Tukey’s post hoc test, *p* * < 0.05, *p* ** < 0.01, *p* *** < 0.001).

Fibroblasts around test materials showing cell adherence were visualized on culture day two by dual staining with DAPI, to identify nuclei and rhodamine-phalloidin to stain actin filaments. The flowable composite was excluded because the fibroblasts did not adhere. The abundant propagated cells around milled acrylic were spindle shaped with positive cytoskeletal and outline staining. Some cells with small cell outlines were present around the bulk-fill composite (Figure 5A). Similar to the WST-1 assay results, cell density increased with cure time, albeit to <15% of the milled-acrylic controls (Figure 5B). The cell density of B80 was significantly higher than that of B20 (*p* < 0.001) and B40 (*p* < 0.01). B60 showed higher cell density than B40 (*p* < 0.05). The cell density of B20, B40, B60, B80 and milled acrylic were 27.78 ± 6.94, 36.67 ± 5.77, 48.89 ± 8.39, 66.67 ± 6.67 and 315.56 ± 25.24 cells/mm^2^, respectively.

### 3.4. Collagen Production

**Figure 6:**
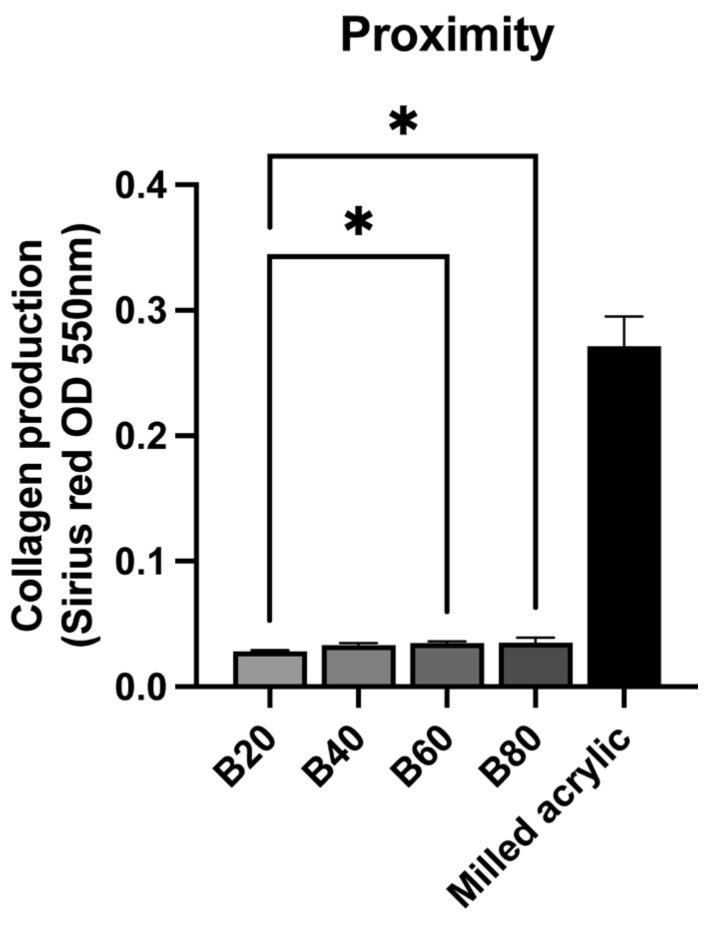
Collagen production by fibroblasts around test materials. Data shown are mean ± SD. Significant differences between test materials are shown (one-way ANOVA followed by Tukey’s post hoc test, *p* * < 0.05).

To evaluate fibroblast function around test materials, Picrosirius-red staining was performed to measure collagen production. The flowable composite was excluded because the fibroblasts did not adhere. Fibroblast collagen production around the bulk-fill composite was <15% of that around the milled acrylic, with production slightly increasing in proportion to cure time (Figure 6). The collagen production of both B60 and B80 was significantly higher than that of B20 (*p* < 0.05). The collagen production of B20, B40, B60, B80 and milled acrylic was 0.028 ± 0.001, 0.033 ± 0.002, 0.035 ± 0.002, 0.035 ± 0.004 and 0.271 ± 0.024 arbitrary units, respectively.

### 3.5. Improvement in Cell Attachment after Surface Removal

**Figure 7:**
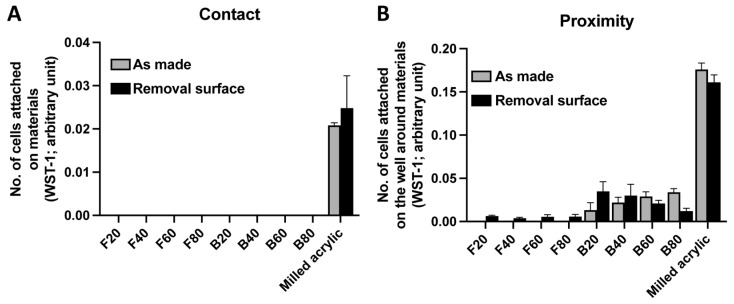
Initial attachment of fibroblasts on and around test materials after surface removal. The number of attached fibroblasts (**A**) on each material and (**B**) around each material. Data shown are mean ± SD.

To determine whether removal of the unpolymerized layer increases the number of attached fibroblasts 2 days after seeding, the WST-1 assay was performed. Regardless of removal, no cells attached to either composite (Figure 7A). A limited number of cells attached around the flowable composite in the proximity experiment. The number of cells around F20, F40, F60, F80 with surface removed were 0.006 ± 0.001, 0.004 ± 0.001, 0.006 ± 0.002, 0.006 ± 0.003 arbitrary units, respectively. An increased number of cells were observed around B20 and B40 and a decreased number of cells around B60 and B80 (Figure 7B). The number of cells around B20, B40, B60, B80 and milled acrylic with surface removed were 0.035 ± 0.011, 0.030 ± 0.013, 0.021 ± 0.004, 0.012 ± 0.003 and 0.161 ± 0.009 arbitrary units, respectively.

## 4. Discussion

Here, we investigated the behavior and function of human gingival fibroblasts cultured with two different composite materials cured for different amounts of time. To model the physical location of cells in relation to the material, the properties of human gingival fibroblasts were evaluated on and around the materials. This allowed us to determine whether the cure time of the materials altered the compatibility of fibroblasts, both in direct contact with and in close proximity to, the materials. In addition, the effect of removal of the unpolymerized surface layer of the materials on initial fibroblast attachment was determined.

In culture experiments, fibroblasts attach to or around the material, and those that do not attach may undergo cell death. We used an indirect method to measure the viability of cells exposed to material cytotoxicity by quantifying the number of cells attached to either the test material (“contact experiments”) or to the culture wells around the material (“proximity experiments”). We studied two types of composite material (flowable composite and bulk-fill composite), with milled acrylic as a control. The milled acrylic was made from a poly(methyl methacrylate) (PMMA) block/disc pre-polymerized at a high temperature and pressure [44,45]. Milled acrylic has the lowest amount of residual monomer and the highest cytocompatibility with fibroblasts of the resin-based materials, so was considered suitable as a positive control [26,46].

In contact experiments, fibroblasts did not adhere to the flowable or the bulk-fill composites after 20 s of curing (recommended by the manufacturer). Both composites had detrimental effects on cell growth. In the proximity experiments, fibroblasts did not adhere in proximity to the flowable composite, but there was some minimal cell attachment around the bulk-fill composite. This suggest that even though composite materials may seemingly have the same clinical properties, apart from viscosity, the cytocompatibility with cells in proximity was substantially different.

Several studies have shown that the cytotoxicity of a composite material depends on its monomer, photoinitiator, and inorganic-filler-particle components [22,47]. In any polymer-based materials, unreacted monomers have negative effects on various cells [10,12,13,16,17,28,33,48,49,50]. Bis-GMA and UDMA are the main components of composites, the former eluting at higher concentrations than the latter [51]. Both monomers cause DNA-strand breaks in fibroblasts, providing a plausible cytotoxic mechanism [52,53]. Flowable composite has a lower filler content and a higher percentage of bis-GMA to reduce viscosity, which might explain our observations of differing cytotoxic effects of the monomers.

We evaluated the behavior and function of fibroblasts cultured with composites for variable curing times of 40, 60, and 80 s. Interestingly, no fibroblasts attached to the flowable composite, regardless of the cure time or physical location. In the contact experiments with the bulk-fill composite, there was similarly no fibroblast adhesion, regardless of the cure time. In the proximity experiments, however, the number of fibroblasts around the bulk-fill composite increased proportionately with the cure time. However, the number of attaching/growing cells was considerably less than that around the milled acrylic. As expected, the number of cells attaching around the bulk-fill composite increased proportionately with the cure time. However, unexpectedly, there was only 20% cell attachment around the bulk-fill composite at a cure time of 80 s, so mitigating cytotoxicity with increased curing is insufficient to solve the cytotoxicity problem.

When composite materials thicker than 2 mm are required, laminate filling is used to compensate for shrinkage during polymerization [54]. We evaluated this up to 80 s, because, depending on the tooth size, the composite resin filled at first may be exposed to light 3–4 times longer than the manufacturer’s recommended time. Further extension of cure time might improve cytocompatibility and reach a plateau, but such long cure times for an intraoral procedure may not be tolerated in practice.

Cells attached around the bulk-fill composite on day 2 had a less well-developed cytoskeleton and were less numerous than those around the milled acrylic, but the number of cells increased on day 4 and day 6. Collagen production also increased in proportion to the curing time. Fibroblasts close to the material were adversely affected, but the curing time did seem to have at least some positive effect in cell proliferation ability and function.

The test materials used in this study were 2 mm thick, and a single irradiation was considered sufficient for polymerization [55,56]. The distance between the light-curing device and the material and its position were fixed, for consistency. Thus, the degree of polymerization at the bottom of the material, furthest from the tip of the light-curing device, was considered to depend on the curing time. As a result, the amount of monomer eluted into the medium varied in proportion to the cure time, and the eluted monomer might have acted on adjacent cells.

The surface layer of composite material does not polymerize when in contact with oxygen, forming an unpolymerized layer due to its polymerization mechanism [13,24,33,35]. We therefore compared composite material with and without the unpolymerized surface layer. Surprisingly, the contact toxicity of both composites did not decrease, despite removing the layer. In proximity experiments, however, removal of the surface layer mitigated the cytotoxicity, but only a few cells attached around the flowable composite. With respect to the bulk-fill composite, the number of cells increased at 20 and 40 s, but decreased at 60 and 80 s. Therefore, removing the surface layer slightly improved the biocompatibility with cells in proximity, but the effect was not proportional to the cure time.

From a clinical point of view, composite materials are often used in the treatment of caries and tooth defect close to the gingival margin. Gingival inflammation and recession of the cervical gingiva are generally considered to be caused by plaque accumulation on the composite materials [57,58]. Hence, differences in bacterial adhesion and biofilm formation due to various curing times and surface treatments should be investigated. In addition, the results of this study suggested that the deleterious effect of composite materials filled in the cervical area may cause cell damage, gingival inflammation, and gingival recession. However, how this cytotoxicity affects the gingival tissue is unclear. Therefore, it is necessary to evaluate host mechanisms or responses to composite materials, for instance by measuring the inflammatory cytokine expression of gingival tissues in direct and close contact with composite materials, in vivo.

The results of this study showed that composite materials are highly harmful to human gingival fibroblasts. A previous study reported that the resistance to negative effects varied among cell types, such as dental pulp stem cells or periodontal ligament cells [59]. There is also a difference between fibroblasts and osteoblasts in terms of their proliferation and differentiation [38]. In addition, many studies have used the experimental method of adding the eluate from the test materials to the culture medium [27,60,61]. The eluate from the material initially releases a large amount of residual components, and then the amount gradually decreases. In our experiment, on the other hand, the test materials were added directly to the culture medium, resulting in the continuous elution of higher concentrations of residual components. Thus, cells could not survive as in the previously reported experiment, due to differences in the resistance of the cells to the material components and to the effects of the different methods of eluting the residual components. Chromatographic analysis is necessary to measure the elution of components over time [18,60].

This study showed that currently used dental-composite materials are non-negligibly cytotoxic. It would now be interesting to assess the host mechanisms or response to the composites, for instance, by the inflammatory cytokine expression of the gingival tissues in direct and close contact with the composite materials, in vivo. The mitigation of composite cytotoxicity with increasing cure time depended on the physical location of the cells, the type of material, and the surface-layer treatment. In light of other potential methods to improve composite materials, N-acetyl cysteine, a precursor of glutathione, the most potent antioxidant in the body, effectively scavenges polymerization radicals and neutralizes chemicals that cause oxidative stress [9,10,15,17,62,63,64,65,66,67,68,69,70,71]. Tri-n-butyl borane, a polymerization initiator, also reduces cytotoxicity, due to the suppressive role of polymerization radicals [11,13,14,18,33,72]. These data provide design rules for the use of monomers and initiators to create novel composite materials with higher biocompatibility. Although this study focused on the effect on gingival fibroblasts, the effects on other cell types routinely exposed to composite materials, such as epithelial cells, whose origin is ectoderm and different from fibroblasts [39,73], and osteoblasts, differentiating cells that uniquely respond to oxidative stress [9,11,74,75,76,77,78], remain to be studied in order to comprehensively assess the biocompatibility of composite materials.

## 5. Conclusions

Here, we examined the behavior and function of human gingival fibroblasts cultured on or around two different composite materials which were cured for different lengths of time. In addition, we examined the effect of removal of the unpolymerized surface layer on cytocompatibility. No cells survived and attached to or around the flowable composite, regardless of cure time. Although no cells attached to the bulk-fill composite, regardless of cure time, some cells survived around the material, and the number increased with longer cure times. Removal of the surface layer slightly improved biocompatibility for cells in proximity, but the effect was not proportional to cure time. In conclusion, the mitigation of cytotoxicity in composites due to an increase in the curing time depends on the physical location of the cells, the type of material, and the finish of the surface layer. These results provide valuable information for clinical decision making and new insights into the polymerization behavior of composite materials. Further in vivo studies are needed to explore changes in biocompatibility at the tissue level.

## Figures and Tables

**Table 1:** Materials used in this study.

Materials (Product Name, Manufacturer)	Main Ingredients	Curing Time (seconds)	Notations
Flowable composite (Aeliteflo™, BISCO Inc., Schaumburg, IL, USA)		20	F20
	Bis-GMA	40	F40
		60 80	F60 F80
Bulk-fill composite (Aelite™ Aesthetic Enamel, BISCO Inc.)		20	B20
	Bis-GMA, UDMA	40 60 80	B40 B60 B80
Milled acrylic (Vivid PMMA Disc, Pearson™ Dental Supply Co.)			
	PMMA	–	–
		

Abbreviations: Bis-GMA, bisphenol A glycidyl methacrylate; UDMA, urethan dimethacrylate, PMMA, poly (methyl methacrylate).

## Data Availability

The data presented in this study are available on request from the corresponding author.
